# The Concerns, Difficulties, and Stressors of Caring for Pets during COVID-19: Results from a Large Survey of U.S. Pet Owners

**DOI:** 10.3390/ani10101882

**Published:** 2020-10-15

**Authors:** Jennifer W. Applebaum, Camie A. Tomlinson, Angela Matijczak, Shelby E. McDonald, Barbara A. Zsembik

**Affiliations:** 1 Department of Sociology and Criminology & Law, University of Florida, Gainesville, FL 32611, USA; zsembik@ufl.edu; 2 School of Social Work, Virginia Commonwealth University, Richmond, VA 23284, USA; tomlinsonc2@vcu.edu (C.A.T.); matijczaka@vcu.edu (A.M.); smcdonald3@vcu.edu (S.E.M.)

**Keywords:** COVID-19, coronavirus, animal welfare, pet owners, human-animal interaction, companion animals, relinquishment, animal behavior, pet owner expectations, dogs

## Abstract

**Simple Summary:**

Relationships between pets and their owners vary greatly. The popular media portrayal of the human benefits of pet ownership is often biased and overstated, oftentimes creating unrealistic expectations for pet owners. There is evidence that pets can be beneficial to the health and well-being of their owners in certain circumstances; however, there is also evidence that negative or ambivalent relationships between owners and pets can be a detriment to owner well-being, as well as a risk for shelter relinquishment (i.e., surrender) of the pet. Relinquishment due to adverse scenarios like the Coronavirus Disease 2019 (COVID-19) pandemic may increase rates of shelter euthanasia of adoptable pets. In this study we identify the unique difficulties related to caring for pets during the COVID-19 pandemic. Pet owners reported concerns related to pets, humans, and the entire household or family, inclusive of people and pets. Data from this study indicate that strength of the owner’s attachment to their pet, as well as their economic resources, and the characteristics of their household are associated with the types of concerns expressed. We discuss implications for human well-being and pet welfare, and the need to address these issues in order to prevent shelter relinquishment.

**Abstract:**

Pets may be a positive presence for their owners during the Coronavirus Disease 2019 (COVID-19) pandemic. However, it is pertinent to identify the hardships associated with pet ownership. We conducted a large-scale survey of U.S. pet owners (*n* = 2254) in spring and summer 2020 to assess the ways that relationships with pets impacted life during COVID-19. We used thematic analysis to analyze 3671 open-ended responses to three prompts. Reported concerns fell into three major categories: (1) pet-focused (meeting needs of pets; procuring supplies; accessing veterinary care; new and emerging behavioral issues; fate of the pet if owner becomes ill; general safety and well-being), (2) human-focused (issues with working from home; well-being and mental health; balancing responsibilities), and (3) household-focused (disease spread; economic issues). Quantitative analyses showed that the owner’s strength of attachment to their pet, economic resources, and relationship status were associated with the types of concerns expressed. Results from this study indicate that pet owners experienced unique hardships related to changes in everyday life from the COVID-19 pandemic. These hardships should be considered alongside the potential benefits found in other studies in order to manage pet owner expectations, prevent pet relinquishment, and more fully understand multifaceted human-companion animal relationships.

## 1. Introduction

Pet ownership is very popular in the United States: recent estimates show that over 60% of U.S. households have at least one pet [1]. News outlets reported a growing popularity in pet adoptions and purchases in late spring and early summer 2020, as the SARS-CoV-2 Coronavirus Disease 2019 pandemic (hereafter referred to as “COVID-19”) spread across the U.S. and stay-at-home or shelter-in-place orders were temporarily issued across most states [2]. Considering that the most effective way to prevent the spread of COVID-19 was to avoid social interactions with people outside of one’s household, pets were considered an especially attractive alternative to fulfill social needs. Some families and individuals may have considered their pandemic work-from-home scenario to be ideal for helping a new pet adjust to their household, as previous work outside of the home would have prevented them from giving pets as much attention and care. New pet owners may have underestimated the amount of work required to care for a pet or could have unrealistic expectations for their health and well-being benefits. Those who already had pets found themselves spending increased time with them while working from home or due to loss of employment. Both new and continuing pet owners may have unmet expectations for their pets to mitigate loneliness and fulfill the social needs usually met by other people. Criticisms of the biased media reporting of research pertaining to the impact of pet ownership on human health and well-being, particularly during the COVID-19 pandemic, point out the danger of setting pet owners up for failure and disappointment, ultimately increasing the risk of shelter relinquishment [3]. There are several reasons why pets may be beneficial and/or detrimental to owner health and well-being during the COVID-19 pandemic [4]. In some cases, the stress of the COVID-19 pandemic may compromise the human-animal relationship and could instigate or accelerate other issues.

### 1.1. Pets and Human Well-Being during COVID-19

Pet ownership is often portrayed as beneficial in terms of physical activity and physical health. Indeed, pet ownership is often associated with increased physical activity, particularly in terms of dog walking [5,6,7,8,9]. Companion animals may also provide stress-buffering benefits for adults and youth; there is emerging evidence of the potential for companion animals to increase oxytocin [10,11,12] and regulate cortisol levels [13,14], thus attenuating the stress-response system (i.e., hypothalamic-pituitary-adrenal (HPA) axis) [15]. Given the growing mental health crisis stemming from the pandemic, another salient aspect of pet ownership is the potential benefits of relationships and bonds with companion animals to mental health, such as loneliness and isolation [16,17,18]. Some empirical evidence indicates that companion animals can provide social support either by acting as a catalyst for social interaction or by offering an interspecies social connection that provides humans with feelings of emotional support and companionship [19,20,21,22]. Companion animal support may be particularly beneficial for children and youth during stay-at-home orders since their ability to interact with peers is limited due to school closures; children often view their pets as confidants and turn to their pets in times of stress or adversity [23,24,25,26,27,28]. 

Equally important to consider in terms of living with pets during COVID-19 are the potential risks of pet ownership. Contrary to research outlined above, other studies found no association between better health and well-being. For example, increased physical activity was not shown to be associated with pet ownership in several studies across various populations [29,30,31,32], and other findings suggest that pet ownership is not significantly associated with improved levels of loneliness in adults [33]. The risk of animal-related injuries (e.g., dog bites) is of particular concern during increased time at home with pets and children [34], and increased time with pets may exacerbate allergy or asthma symptoms [35,36]. Caring for pets with behavioral issues can negatively impact owner well-being [37]. Further, increased time at home also heightens the risk of family violence, of which the family pet may be a victim [38].

The additional stress related to pet ownership (e.g., due to financial strain), potential increases in problematic pet behaviors and concomitant owner frustration, and potential exacerbation of allergy and asthma symptoms from spending increased time at home and around pets may lead pet owners to choose to relinquish their pets [39]. The severance of the relationship via relinquishment during a time of additional stress may further negatively impact the overall health and well-being of individuals.

### 1.2. Pet Relinquishment

In the United States, approximately 6.5 million cats and dogs enter animal shelters each year [40]. Though rates of pet adoption have risen continuously over the last few decades, approximately 1.5 million pets are euthanized in U.S. shelters annually [40]. Relinquishment (i.e., surrender) of family pets is a major contributor to shelter intake and remains an area of focus for intervention as animal welfare professionals aim to reduce euthanasia of adoptable pets. Relinquishment occurs for several reasons, most commonly related to problematic animal behavior, including aggression toward people and other animals [41]. Other common behavioral issues cited for relinquishment include escaping, destructive behaviors, disobedience, excessive vocalization, and excessive need for attention [42]. Indeed, unmet expectations of pet ownership are a common reason for relinquishment, though housing issues tend to be the most frequently cited human factor for surrender [43]. Problematic behavior from pets can be a major stressor that can compromise quality of life [37]; animal outcomes aside, relinquishment could be a relief for the owner in some cases. However, when an owner becomes bonded to a pet, relinquishment can be an unwanted last resort [44].

Strong attachment bonds to pets are considered to be protective against shelter relinquishment [45]; however, socioeconomic factors can often make relinquishment unavoidable [46]. In particular, the U.S. has a shortage of affordable, pet-friendly housing, as pet fees and restrictions are unregulated [47]. In some cases, a strong bond to a pet may actually become a risk factor for housing insecurity, as highly attached owners may refuse to separate from their pet in order to obtain housing [48,49]. Because of these strong bonds and the common regard of pets as family members [50], owners can experience considerable distress upon permanent separation from their pet via death [51] or other unexpected loss [52]. Children may be especially vulnerable to psychological trauma due to animal relinquishment as they are rarely involved in the decision making but may be highly attached to the pet [53].

The COVID-19 pandemic may exacerbate issues with pets for several reasons. As many Americans continue to work from home to mitigate the spread of the virus, pets may be a distraction, particularly in the case of a barking dog. Pets may show new behavioral issues as their schedules and routines change, particularly when owners return to working outside of the home. Many pet-related services have changed or are inaccessible due to the risk of disease spread, so mitigating problematic behaviors can become more challenging. Perhaps most pressing, and of particular concern to animal shelters, is the looming eviction crisis that will displace 30–40 million people from their homes [54]. Combined with the ongoing financial crisis and widespread unemployment, pet owners may have no choice but to separate from their pets if they are no longer able to afford to care for them. Communities will need to identify strategies for keeping pets with their families in order to mitigate adverse scenarios as a consequence of the COVID-19 pandemic. 

### 1.3. The Current Study

It is pertinent to understand the whole picture of human-pet relationships in order to manage owner expectations, prevent problem pet behaviors, improve owner well-being and pet welfare, and ultimately reduce shelter relinquishment. In this study we explore pet owners’ explanations of difficulties, concerns, and stressors related to caring for and living with pets during COVID-19. Given the evidence outlined above that the strength of the attachment bond between the owner and their pet can impact owner well-being and pet welfare (including relinquishment decisions), we then investigated how the owner’s strength of attachment to their pet may be associated with the types of concerns expressed. Additionally, because access to economic resources and characteristics of the household can also impact interactions with pets as well as relinquishment decisions, we also explored how the owner’s income, concern about financial stability, and household composition were associated with the types of concerns expressed.

## 2. Materials and Methods

### 2.1. Data

An anonymous survey was distributed through social media and other online channels (i.e., Facebook, Twitter, Instagram, Reddit, and various listservs) to interest groups and accounts related to companion animals. Responses were collected from 6 April through 21 July 2020, resulting in a convenience sample of 3006 pet owners. Respondents were eligible to participate in the survey if they were at least 18 years of age, residing in the United States, and had at least one pet/companion animal. The survey was only available in English. Survey topics included closed-ended and open-ended questions about respondents’ interactions with pets, sociodemographic characteristics, and changes in homelife, health, and well-being since the COVID-19 pandemic began. Participants also completed a validated scale to assess the strength of attachment to their companion animal(s). Only the screener questions and informed consent were mandatory; therefore, respondents could continue the survey after skipping any question or questions. A total of 2294 respondents completed the survey in its entirety. The survey took approximately 30 min to complete. The study was approved by the University of Florida Internal Review Board, protocol # IRB202000819. The survey instrument is available upon request from the corresponding author.

### 2.2. Sample Characteristics

The majority of the respondents were women (89%) who ranged in age from 18 to 85 years (*M* = 39 years). The majority of respondents (87%) were non-Latinx White; 1% were non-Latinx Black, 2% non-Latinx multiracial, 5% Latinx, and 5% non-Latinx other races. Median family income range was USD 75,000–89,999 per year. Mean level of education was a four-year college degree (33% of respondents), though the mode education was a graduate degree (43%); under 1% reported less than a high school degree, 4% had a high school diploma or equivalent (i.e., GED), 12% had completed some college, and 8% had a two-year college degree.

### 2.3. Measures

Participants were asked a series of closed-ended and open-ended questions related to the stressors, concerns, and difficulties related to caring for and living with pets during the COVID-19 pandemic. They also reported a number of sociodemographic characteristics, including information pertaining to their current economic resources and whether they expected to experience economic hardship due to the pandemic. 

#### 2.3.1. COVID-19 and Life with Pets: Stressors, Difficulties, and Concerns

Respondents were asked the closed-ended question, “Does a pet add stress to the current coronavirus/COVID-19 situation?” Response options were “Yes”, “Somewhat”, “No”, “I don’t know,’ and “Prefer not to say”. Those who responded “Yes” or “Somewhat” (*n* = 407) were presented with the following open-ended prompt: “Please describe how living with a pet has added stress to the current coronavirus/COVID-19 situation”. A total of 374 respondents entered a text response for the prompt; six responses were coded as missing if they did not answer the question directly or gave an uninterpretable response, for example: “Having a pet is stressful”. A total of 368 responses were included in the analysis.

All respondents were asked the open-ended question, “Tell us about any difficulties you have had with your pet(s) by social distancing or shelter-in-place”. Short answers totaled 2099; thirteen responses were coded missing if their response was unrelated to the prompt or uninterpretable. For example, one respondent wrote, “They haven’t been washing their own dishes or giving me advice when I ask for it”. Responses included in the analysis totaled 2086.

All respondents were asked the open-ended question, “What are the pros and cons of living with pets during coronavirus/COVID-19?” A total of 2204 survey respondents completed short answers; 166 responses were coded missing if they entered text unrelated to the prompt, responded in a way that was uninterpretable, or simply wrote “None”. For the purpose of the current study we have omitted responses that did not include cons, resulting in 1217 responses included in the analysis.

#### 2.3.2. Attachment to Pets

Respondents completed the Lexington Attachment to Pets Scale [55], a 23-item measure designed to assess individuals’ emotional attachment to their companion animals. Respondents indicated their level of agreement on a 4-point Likert scale to statements such as, “My pet means more to me than any of my friends”, and “My pet knows when I’m feeling bad”. The summated scores ranged from low to high attachment with potential scores of 32 to 92 (α = 0.90).

#### 2.3.3. Economic Resources

Respondents indicated their total yearly family income for 2019, before taxes, including job earnings, interest or dividends, rent, Social Security, other pensions, alimony or child support, unemployment compensation, public aid, and armed forces or veteran’s allotment. The 26 response options ranged from “Less than $1000” to “$170,000 or higher”.

Survey respondents were also asked to indicate their concern about economic instability due to the pandemic: “Are you worried about losing income due to the coronavirus/COVID-19 situation?” Response options were “Yes”, “Somewhat”, “No”, and “Prefer not to say”.

#### 2.3.4. Household Composition

Respondents indicated their current relationship status by responding to the question, “How would you describe your current relationship status?” Response options were “Married or permanently partnered/cohabiting”, “Single, never married nor permanently partnered”, “Divorced”, “Separated”, or “Widowed”.

Respondents were also asked to indicate caregiver status by responding to the question, “Are you the primary caregiver to anyone?” We determined there was at least one child in the household if the respondent endorsed the response, “Yes, a child or children”.

Additionally, respondents indicated the number of other people in their household by giving a numeric response to the question, “How many people do you live with currently, not including yourself?”.

### 2.4. Analytic Procedures

Three authors with expertise in human-animal interaction coded open responses using a thematic content analysis approach. One author created the codebook for each set of responses based on emergent themes from the data, following a grounded theory approach [56]. To establish intercoder agreement, the percentage of data units that multiple coders have coded identically, two authors coded a random sample of 25–30% of the responses. Upon confirmation of appropriate intercoder agreement (>0.8), one author completed the remainder of the coding for each question. Intercoder agreement value for each code ranged from a low value of 0.88 to a high value of 1.00. Average intercoder agreement value across all codes was 0.96. Intercoder reliability (ICR) was calculated using Cohen’s Kappa, a statistical test of ICR that is sometimes favored over intercoder agreement as it accounts for agreement figures that may have been inflated due to chance [57]. The average Cohen’s Kappa value for our data was 0.69, indicating good overall reliability. Next, qualitative themes were coded into three binary variables and analyzed quantitatively using bivariate tests of association: chi-squared tests were performed for categorical variables, and t-tests for continuous variables. Our data met normality assumptions based on criteria for large samples [58,59,60]. Sample sizes varied for quantitative analyses due to the pattern of missing observations in the dataset; respondents could skip any question. All quantitative analyses were conducted with Stata version 15.1. Qualitative survey responses were analyzed using Microsoft Excel (version 16.40, 2020) and imported to Stata for subsequent quantitative analyses.

## 3. Results

### 3.1. Qualitative Analysis

**Figure 1:**
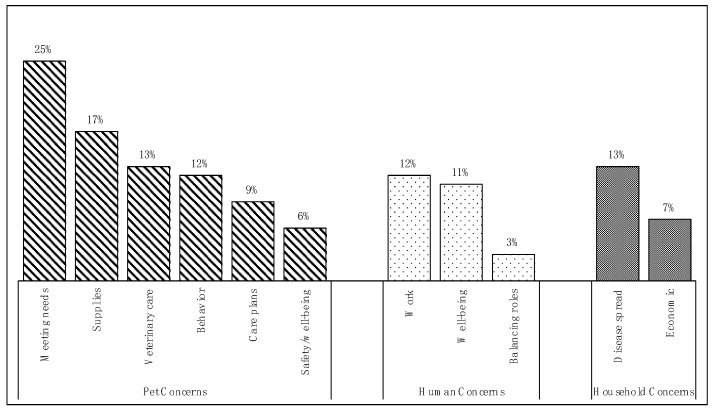
Relative frequencies of themes and sub-themes: concerns, difficulties, and stressors expressed by respondents related to caring for and living with pets during the COVID-19 pandemic.

A total of 3671 responses from 2254 respondents were coded through thematic analysis. Difficulties, concerns, and stressors mentioned fell into three major themes (Figure 1): (1) pet-focused concerns, (2) human-focused concerns, and (3) concerns related to the larger family unit or household, inclusive of both people and pets. Themes were not mutually exclusive; therefore, responses often fell into more than one theme and/or sub-theme.

#### 3.1.1. Pet-Focused Concerns

Pet-focused concerns were most frequently mentioned, overall: 56% (*n* = 1268) of respondents mentioned at least one difficulty, stressor, or con related to caring for and living with pets during the COVID-19 pandemic. The most frequently mentioned sub-theme, coded in 25% (*n* = 556) of all qualitative responses, were issues related to meeting pets’ social and behavioral needs due to changes in everyday life from the pandemic. This included difficulties related to changes in routine due to closures of parks during stay-at-home orders, problems with typical dog walking routes due to increased foot traffic, and concerns related to pets not getting enough stimulation or enrichment due to being confined to the home. One respondent described issues with their young dog: “We have a 6-month-old puppy who is going stir crazy. He’s used to daycare three times per week, lots of social outings, etc. So now he is stuck at home while we are both trying to work and is very demanding of attention”. Some pet owners also mentioned that pets adjusting to increased time together brought out new neediness and apparent expectations of increased attention. Pet owners who had added new pets to their family were concerned about socialization, particularly for puppies: “I have a puppy in the house and am unable to provide the amount of socialization and training that I had planned”.

Respondents mentioned concerns or difficulties related to procurement of supplies for their pets in 17% (*n* = 378) of responses. Pet owners were worried about their ability to get pet food, cat litter, medications, and other various supplies due to businesses being closed temporarily, rumored or actual interruptions in the supply chain, and shipping delays. Some pet owners mentioned anxiety around anticipated supply hoarding behaviors of other pet owners, which would impact their own ability to obtain necessary supplies for their pet(s): “People are hoarding everything, including hay. My horse is a picky hay eater and, because of the hoarding, I can’t get good hay for him”.

Additionally, frequently mentioned were worries related to veterinary care and other pet services, which emerged in 13% (*n* = 297) of responses. Pet owners discussed their concerns with accessing veterinary care when needed, especially for pets with chronic conditions, as well as new protocols at veterinary clinics that do not allow pet owners to accompany their pets during appointments (i.e., curbside drop-off). Euthanasia was a particularly salient concern represented in this theme: “She is old and could develop anytime into a situation that would require her to be euthanized. I am concerned that my vet, because they are not allowing owners to attend their pets’ appointments now, would not allow me and/or my husband to attend a euthanasia inside the hospital. That would make a difficult situation horrible not to be able to be with my cat”. Owners also mentioned concerns related to missed grooming appointments due to closures, as well as trainers being temporarily unavailable or inaccessible.

Related to concerns about missed training appointments were concerns and issues about pet behavioral problems. Twelve percent (*n* = 262) of respondents mentioned concerns related to chronic behavioral problems worsening, new behavioral issues emerging, or the anticipation of new issues due to the pandemic. Most frequently mentioned in this sub-theme were dogs who had developed separation anxiety as they became accustomed to being at home with people all day: “The dog has separation anxiety now if we go outside and leave him alone”. Other owners expressed concern about the expectation that their pet may develop separation anxiety when they return to working outside of the home.

Nine percent of respondents (*n* = 210) mentioned concerns about what might happen to their pet(s) if they were to become ill or incapacitated due to COVID-19. One respondent mentioned both financial and social constraints as limiting factors for a feasible care plan if they were to become ill: “Mostly I am stressed because, if I get sick, I don’t exactly know what I’d do with him. I have a good pet sitter, but at $40/day, it would get extremely expensive for her to look after him for an extended period of time. I don’t have family or many friends around, so I don’t really know who could take him in otherwise”. Relatedly, 6% (*n* = 132) mentioned issues around general safety, welfare, or well-being of their pet(s) during the pandemic. One respondent expressed concern about their pet’s well-being: “He is becoming anxious and losing his social skills with other dogs”.

#### 3.1.2. Human-Focused Concerns

Slightly less than one quarter of respondents (23%, *n* = 516) mentioned concerns, difficulties, or issues related to themselves, their children, or other household members. The most frequently mentioned concern in this theme was related to working from home: 12% (*n* = 269) of respondents expressed work-related issues. Several respondents complained that their dogs interrupted video conferencing by barking, or they found their pet to be distracting during work hours for a variety of reasons. One respondent reflected on their cats and dog both interrupting work: “Our cats can be very vocal when they want stuff, so that can get irritating when working. Our dog barks whenever people walk by, so that gets distracting while working as well”.

Eleven percent (*n* = 248) of respondents mentioned that their well-being was negatively impacted due to increased time at home with pet(s) during the pandemic. Several pet owners expressed irritation, frustration, or annoyance due to their pets’ attention-seeking behaviors, or pets causing disruptions to other activities. One respondent mentioned how frustrating behaviors have become more problematic: “The main difficulty is that my cat is super particular with things being her way, on her own time. It can be annoying during normal times but now it’s amplified being with her all the time. It’s like being with an extremely stubborn person who constantly refuses to compromise”. Some respondents mentioned an increase in their mental health issues as their pet(s) made the situation more stressful, and a few respondents mentioned their difficulty dealing with their pets’ health issues or coping from the death of a pet in isolation. “My dog has some health issues and I worry if something happened to him, I wouldn’t be able to help him. And also, what would I do if something happened to him while shelter(ing) in place—being alone and grieving alone with no comfort”.

A relatively small proportion of respondents (3%, *n* = 59) mentioned difficulties related to balancing pet care with other responsibilities that became more burdensome due to the pandemic. One respondent described stress related to multiple caregiving roles: “Daily feeding, medicating, and cleaning litter are more stressful while caring for (an) at risk elderly parent”. Two respondents specifically mentioned they were experiencing issues related to their children interacting with pets in inappropriate ways.

#### 3.1.3. Household-Focused Concerns

Just under one-fifth of respondents (18%, *n* = 408) expressed concerns related to the family unit or household, inclusive of people and pets. Most commonly mentioned (13%, *n* = 283) were worries related to potential spread of COVID-19, for both pets and people. Respondents described hesitancy to walk dogs in high foot-traffic areas, as well as the burden of shopping for pet food and supplies in addition to groceries. One respondent described many potential infection risks as they went about caring for their pet, and the uncertainty caused by weighing the risk of infection with the need for accessing veterinary services for the safety of their family: “Walking my dog causes me to come in contact with people sometimes. Also, I had to take one of my dogs to the vet, which required going out when we’re supposed to shelter in place. I wasn’t sure what the right thing to do was. Not taking her was a risk, because she had suddenly become aggressive and there is a child in the home, but taking her risked being out and around people”. Other respondents expressed worry about public health guidelines for COVID-19-positive individuals to physically distance from pets in the home.

Seven percent (*n* = 159) of respondents mentioned issues related to their current or impending economic situation. Respondents reported employment loss or changes due to the financial impact of the pandemic and worried they may not be able to afford to care for their pet(s). One respondent noted, “I am worried that as my income becomes less certain (being furloughed/laid off from my job) I won’t be able to afford food, supplies, specialized medication, or veterinary care for all of my pets”.

### 3.2. Quantitative Analysis

#### Pet Attachment, Economic Resources, and Household Composition

On a scale ranging from 32 (low) to 92 (high), the sample reported relatively high average pet attachment scores (*M* = 81.4, *S.D.* = 8.5, *n* = 2101). Mean yearly family income group was USD 60,000–75,000 per year (*n* = 1939); median family income was USD 75,000–89,999, indicating a higher average income than the overall U.S. average. About two-thirds of the sample indicated they were at least somewhat worried about income loss due to COVID-19: 37% answered “Yes”, 30% answered “Somewhat”, and 32% were not worried (*n* = 2093). About two-thirds of the sample were married or partnered (63%, *n* = 1725), 29% (*n* = 791) were single, 6% (*n* = 173) were divorced, 1% (*n* = 34) were separated, and 1% (*n* = 34) were widowed. Less than one-fifth (18%, *n* = 484) of the sample reported that they were the caregiver to a child, and respondents lived with 1–2 other people, on average (*M* = 1.4, *S.D.* = 1.2, range = 0–10, *n* = 2517).

Group differences in pet attachment, income, concern about income loss, relationship status, status of caregiver to a child, and number of people in the household by types of concerns expressed are displayed in Table 1. Mean pet attachment varied by types of concerns expressed: those who mentioned concerns that were coded into the theme “human concerns” had significantly lower (*M* = 79.6) average pet attachment than those who did not (*M* = 81.9, *t*(1853) = 4.92, *p* = 0.000). Yearly family income was significantly lower for those who expressed concerns related to the household or family unit, inclusive of the pet (*M* = USD 50,000–59,000) than those who did not (*M* = USD 60,000–75,000, *t*(1869) = 1.96, *p* = 0.049). Extent of worry for income loss due to COVID-19 varied by types of concerns expressed: Over 70% of those who expressed pet concerns were at least somewhat worried about income loss (*X*^2^(2) = 13.98, *p* = 0.001), and over three-quarters of those who expressed concerns related to their household were at least somewhat worried (*X*^2^(2) = 17.96, *p* = 0.000). Types of concerns also varied by characteristics of the respondent’s household: more than half of those who were married/partnered, single, separated, and widowed expressed pet-related concerns, while less than half of those who were divorced mentioned concerns related to pets (*X*^2^(4) = 18.18, *p* = 0.001). Those who were single most frequently reported human-related concerns, while those who were widowed mentioned human concerns least frequently (*X*^2^(4) = 31.85, *p* = 0.000). Types of concerns expressed did not vary significantly by one’s status of caregiver to a child, nor by the number of people in the household.

## 4. Discussion

In this study we explored hardships, stressors, and difficulties that led to pet owners’ concerns related to caring for and living with pets during the COVID-19 pandemic. We also investigated how types of concerns were related to the strength of the owner’s attachment to their pet, the owner’s access to economic resources, as well as characteristics of their household. Pet owners expressed concerns that fell into three major categories: (1) pet-related concerns, (2) human-related concerns, and (3) household-related concerns. The most frequently cited issues that pet owners reported during the first few months of the COVID-19 pandemic in the United States were related to meeting the social and behavioral needs of their pets. Pet owners discussed having to forgo normal exercise at parks due to closures, rearrange schedules and walking routes due to increased foot traffic, and could not properly socialize or expose pets to everyday experiences. At times, pet owners became exasperated, annoyed, or frustrated by pets’ desire for attention or repeated interruptions during their work-from-home time. Relatedly, pet owners reported new and emerging behavioral issues that their pets displayed due to the changes in everyday life. Most frequently mentioned was separation anxiety, a behavioral issue that has been reported in a variety of species kept as pets [61]. Behavioral issues are the most common reason given for shelter relinquishment [42] and can negatively impact the well-being of the owner [37]. Due to the change in veterinary service delivery (i.e., most veterinary clinics are offering curbside drop-off and do not currently allow pet owners to accompany their pets into appointments), even well-resourced owners may face new barriers to resolving problem behaviors during the pandemic.

As we reported in the Quantitative Results section above, pet attachment scores were significantly lower for those who reported human-related concerns than for those who did not. These human-related concerns included issues related to their own well-being and mental health, problems with working from home with pets, and difficulties balancing roles and responsibilities alongside pet care. Because strong attachment bonds between an owner and their pet are known to be protective of relinquishment [45], this is of particular concern. While this study is cross-sectional and we cannot infer causation, it is possible that the human-animal bond was compromised due to the owner’s issues with the pet during the pandemic. Alternatively, the owner may be less tolerant of frustrations and difficulties with the pet as a result of their weaker attachment bond prior to the pandemic, possibly compromised mental health, and reduced support from people. The COVID-19 pandemic has created a myriad of stressful circumstances related to every aspect of daily life [62]; as our qualitative results reflect, caregiving roles have become more burdensome in many cases. Although the types of concerns expressed did not vary significantly by status of caregiver to a child, nor by number of people in the household, we did find that those who were single tended to more frequently report concerns related to both pets and humans, compared to all other relationship statuses. This could reflect stress related to sole responsibility for pets, for example, the threat of potentially becoming ill or incapacitated due to COVID-19 could be much more significant in terms of responsibility for the care of a pet for those who are single than for those who have a partner to rely on.

Concern about disease spread was mentioned or implied by many of our respondents. This impacted everyday behaviors related to pet care and contributed to issues related to problematic behaviors. This can become especially salient when pet owners weigh their own vulnerability to COVID-19, or that of their household members, with the needs of their pet(s). Several respondents mentioned worry about their pet being vulnerable to COVID-19, or the potential for their pet to spread COVID-19 to vulnerable people. Though few cases of companion animals contracting COVID-19 have been reported to date, and there is currently no evidence of humans contracting the disease from companion animals, worry about disease spread could contribute to relinquishment, abandonment, and killing of pets [63]. Many of the concerns expressed by respondents were similarly characterized by a type of balancing act between human safety and pet welfare. This issue alone could contribute to decreased well-being and heightened sensitivity or lowered tolerance to everyday annoyances and frustrations. Some pet owners may consider alleviating some of their stress by giving up pets and therefore decreasing their caregiving burden.

Only 7% of our sample reported concerns and difficulties related to financial problems; this likely reflects the disproportionately high average socioeconomic status (SES) among our study sample. However, two-thirds of the sample indicated that they were at least somewhat worried about income loss due to the pandemic, indicating some financial uncertainty as the economic consequences of the pandemic remained unclear. Quantitative analyses did reveal that those who reported concerns related to their household, including economic issues, had lower average family income than those who did not. Further, among those who mentioned household concerns, three-quarters were at least somewhat concerned about future economic stability due to the pandemic. The U.S. economic consequences of the pandemic are further exacerbating the already existing wealth disparities [64], and even those who consider themselves to be “middle class” could see new financial stress. Animal shelters are currently bracing themselves for an expected influx of relinquished cats and dogs due to the economic fallout of the COVID-19 pandemic [65]. While the focus of most concerns and mitigation tactics lie in the impending eviction crisis, animal welfare professionals should also be aware of the potential for lifestyle changes due to COVID-19 to impede upon quality of life for pets and their owners, even among those who are economically secure.

### Limitations

This study sample was biased toward non-Latinx White, high-socioeconomic status women as a result of survey recruitment strategies. Though estimates show that non-Latinx White people have the highest rate of pet ownership of any race/ethnicity in the U.S., pet ownership does not tend to vary much by gender, education, or income [1]. A more diverse sample could reveal findings that were not evident with this sample; however, we opted to collect a convenience sample in order to prioritize speed of responses to gather novel information as the COVID-19 pandemic emerged and spread across the U.S. Future studies should explore these research questions in a sample representative of all U.S. pet owners. Comparing the findings presented in this paper with a sample including better representation of minoritized racial and ethnic groups, other genders, and those with lower income could reveal how these issues impact lower-resourced individuals who are already at higher risk of relinquishment due to housing and other economic issues, or lack of access to pet services and veterinary care.

## 5. Conclusions

It is pertinent that animal welfare professionals be prepared to mitigate issues that pet owners are facing during this unprecedented time. Further, some issues may not become apparent until life returns to more-or-less normal, pre-pandemic routines, returning owners to work outside of the home for long days. For example, pets who previously did not display separation anxiety are at risk of becoming destructive and burdensome. Owners should be supported in accessing resources to mitigate any issues that may jeopardize the human-animal bond and increase the risk of relinquishment or abandonment. Especially important are resources and solutions that will be accessible and feasible to people who may be suffering from job loss, economic uncertainty, and housing insecurity. Considering positive relationships with pets may buffer the deleterious effects of stressful or adverse circumstances [22,66], pets could be a source of comfort and normalcy during the pandemic and any resulting fallout, economic or otherwise. Communities can support families and individuals with pets by forming partnerships between human and animal social services in order to meet the needs of the holistic family unit; hence, pet relinquishment prevention is in service of healthy communities.

## Figures and Tables

**Table 1:** Types of concerns expressed by level of pet attachment, yearly family income, worry about income loss due to COVID-19, relationship status, status of caregiver to a child, and number of people in the household.

Group	Pet Concerns ^1^	Human Concerns ^2^	Household Concerns ^3^
Pet Attachment (*n* = 1855)			
“Yes” group ^4^ *M*	81.0	79.6 ***	80.9
“No” group ^5^ *M*	81.7	81.9 ***	81.4
Income (*n* = 1871)			
“Yes” group ^4^ *M*	USD 60,000–75,000	USD 60,000–75,000	USD 50,000–59,000 *
“No” group ^5^ *M*	USD 60,000–75,000	USD 60,000–75,000	USD 60,000–75,000 *
Worried about income loss (*n* = 2013)			
Yes (%)	39% **	37%	45% ***
Somewhat (%)	32% **	31%	31% ***
No (%)	29% **	32%	24% ***
Relationship status (*n* = 2250)			
Married/partnered	55% **	22% ***	18%
Single	63% **	29% ***	20%
Divorced	46% **	12% ***	14%
Separated	55% **	17% ***	31%
Widowed	54% **	4% ***	11%
Status of caregiver to a child (*n* = 2244)			
Has child	56%	21%	17%
No child	55%	23%	18%
Number of other people in the household (*n* = 2071)			
“Yes” group ^4^ *M*	1.3	1.3	1.4
“No” group ^5^ *M*	1.4	1.4	1.4

* *p* < 0.05, ** *p* < 0.01, *** *p* < 0.001, denoting significant group differences. ^1^ “Pet Concerns” refers to the qualitative theme for responses discussing issues related specifically to pets in the household (i.e., meeting needs, procuring supplies, veterinary care, behavioral issues, plans for care if the owner is hospitalized or incapacitated, and safety/well-being). ^2^ “Human Concerns” refers to the qualitative theme for responses discussing issues related to the pet owner or other people (i.e., issues with work, well-being, and balancing roles). ^3^ “Household Concerns” refers to the qualitative theme for responses discussing issues related to the household unit, inclusive of pets and people (i.e., disease spread, economic issues). ^4^ “Yes” group indicates that the respondent mentioned concerns related to the corresponding theme. Figures in cells reflect group means. ^5^ “No” group indicates that the respondent did not mention concerns related to the corresponding theme. Figures in cells reflect group means.
